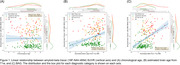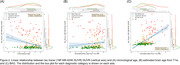# Brain Age Gap Correlates with 18F‐NAV‐4694 and 18F‐MK‐6240 Standardized Uptake Value Ratio

**DOI:** 10.1002/alz.093282

**Published:** 2025-01-09

**Authors:** Reza Rajabli, Mahdie Soltaninejad, Neda Shafiee, Vladimir S Fonov, Nesrine Rahmouni, Stijn Servaes, Joseph Therriault, Serge Gauthier, Pedro Rosa‐Neto, D Louis Collins

**Affiliations:** ^1^ McConnell Brain Imaging Centre, Montreal Neurological Institute, McGill University, Montreal, QC Canada; ^2^ Translational Neuroimaging Laboratory, The McGill University Research Centre for Studies in Aging, Montréal, QC Canada; ^3^ Douglas Mental Health University Institute, Montreal, QC Canada; ^4^ Translational Neuroimaging Laboratory, The McGill University Research Centre for Studies in Aging, Montreal, QC Canada

## Abstract

**Background:**

It is feasible to train a model on a healthy cohort to estimate the chronological age from a T1‐weighted (T1w) MRI. This model can be used to estimate the apparent brain age of subjects with Alzheimer's Disease (AD). The difference between the true chronological age and the apparent brain age, called Brain Age Gap (BAG), is a potential feature to estimate the level of pathology and neurodegeneration of an individual patient with AD. To further study this, here we examined the linear relationship between BAG and the standardized uptake value ratio (SUVR) of the amyloid‐beta tracer (18F‐NAV‐4694) and tau tangle tracer (18F‐MK‐6240).

**Method:**

We used ∼40K T1w MRIs from the UK Biobank dataset, with improved preprocessing and more extensive data augmentation, to train an SFCN‐reg model (Leonardsen 2022). Our implementation achieved a generalization gap error of less than 1 year, surpassing the performance reported in the original study. Then, using the trained model, we estimated BAG for 245 T1w images from the Translational Biomarkers in Aging and Dementia (TRIAD) dataset (Therriault 2022) consisting of 146 normal controls (NC), 46 with Mild Cognitive Impairment (MCI) due to AD, and 53 with AD dementia. Subsequently, We compared the estimated BAG values with the SUVR values for neocortical amyloid and tau PET meta‐ROI (Jack Jr. 2016).

**Result:**

Figure 1 illustrates the linear relationship between the 18F‐NAV‐4694 SUVR for all subjects (at baseline scan) vs age (no correlation), apparent brain age (r = 0.31, p << 0.001), and BAG (r = 0.43, p << 0.001). Figure 2 illustrates the linear relationship between the 18F‐MK‐6240 SUVR vs age (r = ‐0.27, p << 0.001), apparent brain age (r = 0.11, p < 0.08), and BAG for the same subjects (r = 0.58, p << 0.001).

**Conclusion:**

We demonstrated a meaningful relationship between 18F‐NAV‐4694 and 18F‐MK‐6240 SUVR values and BAG, stronger than with age or apparent age, reinforcing the notion that BAG can serve as a feature to estimate the amount of neurodegeneration due to accumulation of amyloid and tau when more precise data, such as PET scans, are not available.